# Disabled people’s experiences of accessing reasonable adjustments in hospitals: a qualitative study

**DOI:** 10.1186/s12913-018-3757-7

**Published:** 2018-12-04

**Authors:** Stuart Read, Pauline Heslop, Sue Turner, Victoria Mason-Angelow, Nadine Tilbury, Caroline Miles, Chris Hatton

**Affiliations:** 1 Norah Fry Centre for Disability Studies, School for Policy Studies, University of Bristol, 8 Priory Road, Bristol, BS8 1TZ UK; 2 National Development Team for Inclusion, First Floor, 30-32 Westgate Buildings, Bath, BA1 1EF UK; 3 Faculty of Health and Medicine, Furness College, Lancaster University, Lancaster, LA1 4YG UK

**Keywords:** Disabled people, Reasonable adjustments, Equality act 2010, Hospital care

## Abstract

**Background:**

The UK Equality Act 2010 requires providers of health services to make changes or ‘reasonable adjustments’ to their practices in order to protect disabled people from discrimination or disadvantage when accessing care. Existing evidence suggests that despite this legislation, health services are not always providing reasonably adjusted care for disabled people. This paper presents the perspectives of disabled people themselves in relation to their experiences of accessing reasonable adjustments in hospitals in England.

**Methods:**

Twenty-one semi-structured interviews were held with disabled people who had a recent experience of hospital care in England. Participants were asked about the extent to which the hospital provided reasonably adjusted care, and if necessary, how they thought the provision of reasonable adjustments could be improved. Each interview was anonymised and transcribed, and the data analysed using thematic analysis.

**Results:**

Participants reported mixed experiences about whether and how reasonable adjustments were provided: some shared positive examples of good practice; others spoke about difficult encounters and limited provision. Recommendations made include a need for culture change in how reasonable adjustments are perceived and enacted; improvements in identifying the needs of disabled people; improvements to the hospital environment and the provision of information; and the need to involve disabled people themselves in the process of change.

**Conclusions:**

Gaps remain in how reasonable adjustments are provided for disabled people accessing hospital care. It is important for hospital staff to listen to the perspectives of disabled people about the provision of reasonable adjustments, and make improvements as necessary. Hospital staff could also do more to share good practice in relation to the provision of reasonable adjustments to effectively inspire and embed positive change.

**Electronic supplementary material:**

The online version of this article (10.1186/s12913-018-3757-7) contains supplementary material, which is available to authorized users.

## Background

Within England, healthcare providers are required to ensure that the care they provide to patients is ‘person-centred’, and designed to suit each person’s individual needs, wishes and preferences [1]. Person-centred care is achieved in practice through the patient and healthcare provider working collaboratively to achieve desired care strategies and outcomes, rather than the patient being a passive recipient of care [2, 3]. Person-centred care has been successful in demonstrating improvements in health outcomes [4].

An additional dimension of person-centred care for disabled people^1^ is ensuring that they have full access to health services – and this is achieved through the provision of ‘reasonable adjustments’^2^ [1, 5]. The UK Equality Act 2010 [6] requires public services, including health services, to provide changes or ‘reasonable adjustments’ to their practices to ensure that disabled people are not denied access to the quality of care afforded to non-disabled patients. The requirement is ‘anticipatory’, so services are required to anticipate and make provision for adjustments that disabled people may require (S149) [6, 7]. The Equality Act 2010 [6] defines a disabled person as anyone who has ‘a physical or mental impairment’ and for whom the impairment has ‘a substantial and long-term adverse effect on [their] ability to carry out normal day-to-day activities’ (S6.1).

The Equality Act 2010 [6] directs that services must consider the provision of reasonable adjustments in three ways. The first is changing the physical features of a service so that disabled people can access it, such as making buildings wheelchair accessible, or changing the visual appearance of wards or departments (e.g. making them ‘dementia friendly’ [8]). The second is changing existing practices or procedures to make access easier for disabled people, such as changing the timing, length or location of a disabled person’s health appointment [5]. The third is providing auxiliary services or aids so that disabled people are better able to access healthcare, such as providing a British Sign Language interpreter [9], or providing information in accessible formats [10]. These examples highlight that the provision of reasonable adjustments by healthcare providers can involve making global changes to health services that will benefit many disabled people, but also changes at an individual level to help meet the specific needs of a disabled person.

There are a number of recent initiatives in England to encourage reasonably adjusted care for disabled people in hospitals. The National Health Service (NHS) Standard Contract requires providers of health services to comply with the Equality Act 2010 [11]. The Accessible Information Standard [12] aims to ensure that disabled people are able to communicate and to access information in a way that is suited to their individual needs. NHS Digital [13] is working to develop a ‘flag’ on the Summary Care Record, which is a collection of a patient’s individual information created from their medical records held by their general practitioner. This will alert staff that the person is entitled to the provision of reasonable adjustments, and provide a record of the exact adjustments agreed. Finally, a series of documents detailing the type of reasonable adjustments needed for people with intellectual disabilities living with different healthcare conditions has been produced by Public Health England [14].

Despite the legal requirement and policy initiatives to provide reasonable adjustments for disabled people, evidence suggests that they are not being consistently provided by hospital services [7, 15, 16]. Many NHS Trusts (public bodies with responsibility for the provision of health services in a geographical area) have demonstrated a commitment to providing effective reasonable adjustments for disabled patients, such as ensuring staff receive training about the needs of disabled people [7]. However, research by Tuffrey-Wijne and colleagues [15, 16] suggested that although legislation or policy may be in place, the responsibility to enact it lies with individual hospitals. Therefore, whether and how reasonable adjustments are provided is shaped by the knowledge and responsiveness of hospital staff, and the resources that the hospital is willing to provide to ensure reasonably adjusted care. This is an important point to consider, as what reasonable adjustments are and how these are enacted may not be well understood by care providers [15–18].

Disabled people have reported barriers when accessing health services, including difficulties navigating inaccessible environments [19]; and inconsistent use of flagging systems, accessible information, and hospital passports (documents that are often used with people with intellectual disabilities to provide hospital staff with important information about their individual needs and health when they are admitted to hospital) [7, 15, 16, 20, 21]. Together, this raises questions about the quality of care that disabled people can expect to receive [17], which in turn, can affect their health outcomes, for example, a lack of reasonable adjustments was reported to be a contributory factor to the premature deaths of people with intellectual disabilities [22, 23].

Evidence relating to the provision of reasonable adjustments by hospitals commonly focusses on patients with intellectual disabilities; evidence relating to disabled people with a broader range of impairments is more limited. In addition, disabled people’s own recommendations about how hospital services could potentially improve the provision of reasonable adjustments has rarely been sought. The aim of this study was to investigate first, disabled people’s own experiences of the provision of reasonable adjustments by hospital services; and secondly, to explore their ideas about potential improvements.

## Methods

### Materials

We conducted semi-structured interviews with disabled people about their own experiences of receiving reasonably adjusted hospital care. To develop the project information materials and topic guide, we worked with an advisory group of disabled people. The group suggested structuring the interview in a way that allowed disabled people to share their hospital ‘journey’, exploring their experiences before they arrived at hospital, during their hospital visit, and when they left hospital. The topic guide for the interviews was therefore developed specially for this research study and structured in this way see Additional file 1.

### Recruitment

To participate in the study, we required people to personally identify as disabled; be aged 18 years or older; and have accessed hospital care (e.g. inpatient, outpatient or accident and emergency) in England for a personal health need within the past 2 years. Information about the study was cascaded to disabled people via networks of disability and health or self-advocacy organisations in England. Disabled people interested in participating in the study contacted the research team to learn more about the project. They were then sent a study information sheet and a consent form to look at, and asked to think about one hospital experience in the past 2 years that they would feel comfortable discussing with the researcher. On the day of the interview, the interviewer discussed the project information sheet and consent form with the disabled person to ensure that they understood the interview process, and that they were happy to continue.

### Procedure

Interviews were conducted either in person at the participant’s home, or via telephone. The structure of the interview followed the topic guide, and focused on understanding participants’ hospital experiences, and whether and how reasonable adjustments had been provided, if required. Participants were asked whether the provision of reasonable adjustments could be improved by hospitals, and if so, in what ways. Each interview lasted approximately 1 h, and was audio-recorded, with consent.

The study received ethical approval from the Faculty of Social Sciences and Law Committee for Research Ethics, University of Bristol in 2016 (reference 30501).

### Analysis

Interviews were transcribed and the transcripts anonymised. We used thematic analysis as an analytical framework, as described by Braun and Clarke [24]. Using NVivo 10 software, the lead author independently read all of the interview transcripts and assigned codes to establish patterns from the texts. Themes and linked subthemes were then extracted. Through a process of iterative reading and analysis, codes and themes were modified and shaped, and an initial coding frame based on the thoughts and reflections of the lead author was produced. Each of the interview transcripts were then independently read by two co-authors, who recorded their initial thoughts for emerging codes and themes. The three researchers then met to collaboratively discuss ideas about the data and to agree the final themes relating to disabled people’s experiences of reasonable adjustments to the hospital care they received, and to their recommendations for improvements.

## Results

Twenty-one disabled people participated in the study. As Table 1 shows, this included 12 women, eight men, and one couple (one man and one woman). Participants were drawn from across England.

**Table 1:** Participant characteristics

Participant Number	Gender	Region	Method of Interview
1	F	North East	In person
2	F	North East	In person
3	F	North West	In person
4	F	West Midlands	In person
5	F	East Midlands	Telephone
6	F	South West	In person
7	F	South West	In person
8	F	South West	In person
9	F	South West	Telephone
10	F	South East	In person
11	F	London	In person
12	F	Missing	Telephone
13	M	North East	In person
14	M	North East	Telephone
15	M	East of England	In person
16	M	East of England	In person
17	M	South West	Telephone
18	M	London	In person
19	M	London	Telephone
20	M	London	Telephone
21	M and F couple interviewed together	East Midlands	In person

Although the study did not require disabled participants to disclose the nature of their impairments, the content of many of the interviews indicated that participants experienced a range of different impairments including physical impairments, sensory impairments, intellectual disabilities, and mental health support needs.

Five themes relating to reasonable adjustments to the hospital care disabled people received were identified from the interview data: (i) the process of identifying a person’s need for reasonable adjustments; (ii) reasonable adjustments in relation to the physical features of a hospital; (iii) changes to existing practices within a hospital; (iv) the provision of additional aids or services; and (v) recommendations for the provision of reasonable adjustments for disabled people by hospitals.

### The process of identifying a person’s needs for reasonable adjustments

Participants discussed a variety of ways in which their need for reasonable adjustments was identified and then recorded or ‘flagged’ on hospital systems. Identifying that a person is disabled and may need reasonable adjustments usually precedes the more formal process of ‘flagging’, whereby a hospital alert or ‘flag’, is placed on the disabled person’s records to remind professionals that reasonable adjustments are required [13]. The distinction between identification and ‘flagging’, however, lacked clarity for most participants.

One participant (P7) with intellectual disabilities described a positive experience of a health professional reviewing her hospital passport with her, commenting that *‘it’s good having it’* to ensure that the staff understood her needs. However, this was an uncommon experience amongst the participants. More frequently, participants indicated that their needs were not identified and recorded, as the following exchanges typified:

*Interviewer: did the hospital do anything that you can think of that made them aware about your needs? So, did they perhaps contact you to talk about your needs, and what adjustments may be provided?*

*Participant: No, there's never any...I've never had any contact of that nature.* (P4)

*Interviewer: As you have been to hospital a few times, were your needs actually flagged up on the system? You said that you were well known to the staff.*

*Participant: To be honest, I'm finding I'm having to explain what I can do and I can't do to the nurses* (P18).

### Reasonable adjustments in relation to the physical features of a hospital

Participants made many references to the need for, or provision of reasonable adjustments in relation to the physical features of a hospital, such as its physical accessibility for people with mobility or sensory impairments. Some participants spoke about positive changes to a hospital’s physical features to make the environment more accessible for disabled people – sometimes in a number of creative ways. For example, one woman noted how the outpatient department was accessible to disabled people with different impairments:

*…everything is on the flat […] they're nice, wide corridors actually. And they're well lit. And they do have hand rails. Which are a great help, you know. Especially if you're not too steady on your feet, it's always nice to know there's something there to grab hold of, if you happen to be walking. And […] that's a low-level desk, so that you have no trouble, you're not straining or anything, you know. And as far as I'm aware, they have induction loops for if you were wearing […] hearing aids* (P9).

Other participants had mixed experiences about the extent to which the hospital had adjusted its physical features, for example noting that although some changes had been made, they were insufficient to address the barriers experienced by disabled people. One man with a physical impairment explained that although some physical features of the hospital were suitable, he was at a disadvantage because of an inaccessible door release:

*The corridors were wide enough. Again, using the lifts, the buttons are at a reasonable level. There's two – there's three floors. There's an announcement in the lift, there's Braille on the lift buttons, there's signage. The doors, because obviously with security they have to remain closed, but I found that when I was using – when I was in my powered wheelchair, I was limited to going places. I had to wait for someone to come and open the door […] I mean other people were using it quite normally and pressing the button to let themselves out. Whereas I couldn't reach it. Had to call a member of staff, or someone to come past and ask them to push the button to release the door, for them to open the door* (P18*).*

Other participants described difficulties trying to use hospital services, suggesting that little or no attention had been paid to identifying if a person was disabled and required reasonable adjustments. For example, one woman (P5) described difficulties when attending a mammogram appointment, when the cubicle was too small for her to be able to get changed easily.

### Changes to existing practices within a hospital

Some participants stated that hospital staff were willing and able to adjust standard hospital practices to meet their needs. The examples shared by participants indicated that such reasonable adjustments had been provided both to help disabled people engage with the hospital system as a whole, and with specific procedures. For example, one participant with a physical impairment described how his doctor understood and supported his request to have his wheelchair with him:

*When I was in hospital, after a day, they said my condition was quite bad, and sent me to the intensive care unit, where there was an issue with having a powered wheelchair near the equipment. Which I can understand. And thankfully the doctor said, 'No, he needs his wheelchair.' Because I said to the doctor I – you know, I function better when I'm sitting up* (P18).

Another participant with a visual impairment, explained that health professionals had understood and accommodated his needs effectively:

*The consultant knew that I couldn't see. So sometimes she would guide me to the couch to lay on, so she could do....... And she was...you know, she explained what she was doing […] she explained everything* (P17).

Such positive experiences were not described by all participants and some talked about how their needs as a disabled person were not met. For example, one woman said:

*…I need an MRI* [Magnetic Resonance Imaging] *scan and then you go in one of these things, and I tell them (health professional), like ‘hey, I have hearing impairments, can you please stand on, you know, that side of my head?’ […] and then when you’re in there […] They sit on the wrong side or they, you know, gotta talk very loud, and I’m like, ‘no, that’s not gonna really help me’* (P12)

### The provision of additional aids or services

Participants reported many examples of whether and how a hospital had provided additional aids or services for them. Key issues were accessible information, hospital transport, and the provision of additional assistance. In some cases, participants reported that they had been supported in a positive way by the hospital; others reported that their needs were not met appropriately.

One participant, for example, reported that she was provided with appropriate information, saying *‘I did have a lot of information sent to me, yeah. […] It was all easy language, all easy words’* (P7). Other participants, however, reported that the hospital did not appear to make accessible information readily available. One person with a visual impairment explained: *‘I prefer text, phone call or maybe emails […] but they do prefer to [send] - just a letter […] and that’s not appropriate for me*’ (P20).

A variety of experiences was also reported in relation to hospital transport. One person noted how she was pleased with the hospital transport service:

*…we've got a very good hospital transport service. And if I haven't been able to get there, or if I have been in my wheelchair, and I've got a hospital appointment, I phone up and I get a hospital transport ambulance to come and get me, and I'm taken in to the appointment* (P9).

Others reported disappointing experiences, including transport being provided that was not accessible for people using a wheelchair:

*They sent an ambulance out, ambulance people say, ‘No, you can't take your wheelchair. Can't take your manual chair at all. It's an emergency ambulance, you can't have the wheelchair in it.’ So, I'm thinking, ‘Well I'm going to be a nightmare to nurse if I haven't got a chair at all up there. What's going on here?’ So, I had to get hold of one of the people that work for me and ask her if she'd come and collect a wheelchair to take it up to the hospital, so I had a manual chair to be in the ward* (P3).

Another key area of concern to the disabled participants was having assistance at appointments. Again, participants reported mixed experiences. One participant commented: S*omebody helped me, you know. You know, took my arm and things like that […] [they] asked which side I wanted, you know, which way did I want to go, left or right, things like that* (P13). Another participant commented:

*The radiographer did [provide assistance]. I told her I couldn't see where I was going, she led me. She did everything she should have done. She was very patient-sensitive, if you like. But nobody else in the hospital was* (P5).

### Recommendations for change

Participants shared many ideas about the ways in which they thought improvements could be made to the provision of reasonable adjustments for disabled people by hospitals. Five key recommendations were made: a) culture change in how reasonable adjustments are perceived and enacted; b) improvements in identifying the needs of disabled people; c) improvements to the hospital environment; d) improvements to the provision of information; and e) disabled people themselves being involved in the process of change.

#### ‘Culture change’ in how reasonable adjustments are perceived and enacted

The most commonly reported recommendation was the need for ‘culture change’ within the NHS in terms of how reasonable adjustments are perceived and provided by hospital staff. Participants generally understood and described ‘culture change’ as being related to the ways in which staff values or attitudes had an impact on their practice. The participants recommended that hospital staff should be more aware, open and responsive to the need for reasonable adjustments for disabled people. Strategies to bring about ‘culture change’ were proposed at a range of levels, including staff taking time to listen to disabled people themselves; the provision of staff training about the needs of disabled people; and systems and processes to be in place to clearly record a disabled person’s needs. As one participant commented: ‘*I don’t want special. I just want appropriate. […] But that means listen to people’* (P3).

#### Improvements in identifying the needs of disabled people

Another commonly reported recommendation for change was to better identify and record the needs of disabled people. Participants suggested the recording of a person’s needs on a personalised form such as a hospital passport, or on a standard reporting form currently in use throughout the hospital. One participant commented:

*Well I think what they could have done is that on – considering the reception probably would have had a form for me […] they could have probably had a section on there as to what needs I had. Like, you know, needs a wheelchair […] you know, sort of take the initiative.* (P14).

This was echoed by another interviewee, who considered that improvements to how a person’s needs were recorded would be beneficial to the overall running of the hospital:

*You know, I did talk to them about it, and I said, 'You haven't got enough boxes to tick, like, ‘This patient needs a carer with them.’ ‘This person's in an electric wheelchair.' If those boxes were ticked, then appointments wouldn't be made that were wrong* (P21).

#### Improvements to the hospital environment

The third key area for recommendations was for improvements to hospital environments. Recommendations were wide-ranging, and addressed a number of areas including improving wheelchair accessibility or the general physical access of a hospital; ensuring that disabled parking was close to the entrance; making sure that hospital transport services were accessible and can carry a support worker or carer if required; the provision of equipment or support to assist disabled people, such as hoists and hearing loops; and paying attention to signage, colour and general visual accessibility of the hospital so that disabled people are able to navigate their surroundings easily.

#### Improvements to the provision of information

Disabled people also recommended that improvements are required to the way in which information is provided by hospitals. Several spoke about the importance of receiving information that was in an accessible format for their needs such as large print, or easy-read materials, or information provided using different formats such as on a CD instead of using print. Hospital systems should be able to identify the specific information needs of disabled people and respond to them appropriately.

#### Disabled people themselves being involved in the process of change

Finally, some participants recommended involving disabled people themselves in identifying local opportunities for change and providing advice about implementing change. One participant (P16) recommended asking disabled patients to complete satisfaction questionnaires, or to take photographic evidence of any barriers they encountered to prompt service improvements. Others stressed the importance of disabled people themselves shaping improvements. For example, one participant who worked as a volunteer in her local hospital (P2) illustrated how being ‘on the ground’ could enable small but significant changes that increased accessibility for disabled people. She had identified that some automatic doors were problematic for disabled people as they were ‘swinging out too quickly’, so she reported this to the maintenance team and within a week the speed of the door opening had been changed and slowed down. It was the fact that she was able to identify this as a disabled person herself, and knew who to report it to, that appeared to have been instrumental in getting this changed.

## Discussion

This study explored disabled people’s own experiences about the provision of reasonable adjustments by hospital services, and their ideas about how this could be improved, if indicated. Participants reported mixed experiences about how reasonable adjustments were provided: some shared positive examples of good practice; others spoke about difficult encounters and limited provision. Challenges were in relation to the process of identifying a person’s need for reasonable adjustments; the physical features of a hospital; existing practices within a hospital; and the provision of additional aids or services.

In 2008, the Michael Inquiry reported that ‘There is a clear legal framework for the provision of equal treatment for people with disabilities and yet it seems clear that ... services are not yet being provided to an adequate standard’ ([25]: p.55). The legal framework was strengthened by the Equality Act 2010 [6], but the Life Opportunities Survey [26], a large-scale longitudinal survey of disability in Great Britain which was conducted at about the same time as the introduction of the Equality Act 2010, reported that up to 13% of disabled adults identified health staff as being responsible for discrimination they experienced. Disabled adults were significantly more likely than their non-disabled peers to experience barriers in accessing health care to the extent that ‘health care provision in Great Britain is failing to meet its statutory requirement to provide ‘reasonable adjustments’ to ensure equality of access for disabled adults’ ([19]: p.926).

Such concerns are not restricted to England. In the years following the passing of the Americans with Disabilities Act in 1990 [27], there was a growing body of research suggesting that barriers to healthcare access were persisting for disabled people because of the lack of reasonable adjustments provided [28, 29]. A range of structural, financial, cultural and personal barriers to accessing healthcare were reported by disabled people of different ages and with a wide range of impairments, findings which were consistent with other studies carried out nationwide [28].

Research evidence about the identification and provision of reasonable adjustments by hospitals in England for disabled people subsequent to the Equality Act 2010 is scant but suggests the variable provision of reasonable adjustments by hospital services similar to that reported by disabled people in this study. A survey of 119 hospital Trusts (30% of all NHS Trusts in England) [7] concluded that some forms of reasonable adjustments were being delivered in many Trusts, particularly relating to the provision of accessible information and the use of hospital passports. The authors also identified that far fewer Trusts provided evidence about the provision of reasonable adjustments relating to the face-to-face treatment of patients. Our study participants did not identify a divide between collective adjustments for groups of patients and individualised adjustments for a particular patient, possibly because they were reflecting on the quality of provision provided to them, as well as the availability of the adjustments.

Tuffrey Wijne et al. [16] researched factors that promote and compromise the provision of reasonably adjusted healthcare for patients with intellectual disabilities in NHS hospitals. They reported that in order for reasonable adjustments to be embedded, hospital staff must be allowed to identify when disabled people require reasonably adjusted care, and provided with the necessary management support and resources to deliver the adjustments [15, 16]. Effective collaboration between staff and departments is another important factor [15, 16, 22, 23]. Our study participants reflected on times when there was no consistent identification and recording of their needs, which resulted in them repeatedly having to retell their ‘story’ and request adjustments to their care. Generally, they had felt disempowered by this, and some reported that such occasions had made them feel a ‘nuisance’. The overwhelming impression was that the provision of reasonable adjustments was on an ad hoc basis in response to a direct request and dependent on the largesse of an individual staff member. Were hospital procedures in place that could easily identify disabled people and the adjustments they required, and staff were equipped with the authority to deliver the adjustments, some of the problems faced by the participants may have been avoided.

Ward culture and staff attitudes are ‘crucial’ in ensuring that hospital services are accessible ([16]: p.1). The need for ‘culture change’ was the most commonly reported recommendation from the participants in our study, but the views of the participants about how this should be brought about differed from research based on the perspectives of hospital staff. Our study participants advocated staff taking time to listen to disabled people themselves, the provision of staff training about the needs of disabled people, and systems and processes to be in place to clearly record a disabled person’s needs – all issues close to their experiences as disabled people accessing healthcare. A large scale study of culture and behaviour in the NHS in England [30] summarised strategies for creating positive cultures as listening to staff and encouraging them to be involved in decision making, problem solving and innovation; providing staff with helpful feedback; taking effective, supportive action to address system problems when improvement is needed; fostering good teamwork; and ensuring that staff feel safe, supported, respected and valued at work. Perhaps we should add the word ‘patient’ to the word ‘staff’ in the summary above to emphasise that a culture in which patients as well as staff feel listened to, involved in decision-making and respected and valued is a culture which will work well for both patients and staff.

The good practice examples shared by disabled people in this study highlight the need for hospitals to review, share, and learn from such examples, including the strategies used to enact them, in order to promote and evidence how hospitals provide reasonably adjusted care, and to help embed positive change [5, 31].

Participants in our study recommended involving disabled people themselves in service improvements. When Trusts review how reasonable adjustments are provided in their services, they must focus on listening to, and understanding, the perspectives and experiences of disabled people themselves [10, 15, 20, 22, 23]. For disabled people’s insights to contribute to meaningful service change, hospital services need understand the importance of working cooperatively with disabled patients [32].

Most of the existing research about the provision of reasonable adjustments by hospitals relates to people with intellectual disabilities [7, 15, 16, 20, 21], what is original about our paper is that our research builds on these findings from a pan-disability perspective. In 2008, the Department of Health (England) stated that ‘if services and health outcomes are improving for people with learning disabilities, they are likely to be improving for other groups at risk of health inequalities’ ([17]: p.6). Learning from the experiences of people with intellectual disabilities can therefore provide a ‘benchmark’ for the care of other disabled people accessing hospital care.

Our study clearly highlights that research about the provision of reasonable adjustments by hospitals for disabled people is a significant issue requiring future research. Specific aspects that future research could cover include: i) the proportion of the patient population that requires the provision of reasonable adjustments; ii) the most commonly required adjustments needed by hospital patients and their cost; iii) the input required by hospital staff, systems and processes, and their cost, to ensure that hospitals are consistently and effectively meeting the requirements of the Equality Act 2010, in particular the anticipatory duty to make reasonable adjustments.

### Strengths and limitations

There are a number of strengths and limitations of this study. One strength is its consideration of different impairment groups in the provision of reasonable adjustments. The Equality Act 2010 and its concept of reasonable adjustments is not disability-specific, so exploring the commonalities and differences across different impairment groups can be instructive. Another strength of the study is the inclusion of the voices and experiences of disabled people and their recommendations for change.

There are some potential limitations to the study too. All participants identified themselves as disabled, but research into attitudes towards, and experiences of, disability has shown that disabled people vary as to whether they perceive themselves to be ‘disabled’ or not. The Office for National Statistics Opinions Survey in 2012 [33] included a question asking those who came under the Equality Act 2010 [6] definition if they thought of themselves as disabled, and 62% did not. Mont [34] suggests that the self-identification of disability generates the lowest prevalence rates of disability. Further potential limitations are that participants were recruited via existing disability and health organisations, and all participants were able to verbally report their views and experiences with little help. The consequence of these potential limitations is that the experiences described in this paper may come from relatively independent disabled people who have a particular interest in highlighting or changing health care practice, or who feel empowered to ensure that they receive reasonably adjusted care. Although we tried to ensure diversity of participants, such as in terms of gender, region and impairment, their stories cannot be considered representative of all disabled people’s experiences. Further, all participants were asked to describe an experience of accessing hospital care from within the past 2 years. Their experiences may therefore not reflect recent initiatives to improve the provision of reasonable adjustments for disabled people in hospitals.

## Conclusions

In England, hospitals are required to make reasonable adjustments for disabled patients accessing care. Some disabled participants in this study reported evidence of effective reasonably adjusted care, but this was not common, and the overall picture was mixed. Gaps remain in how reasonable adjustments are provided for disabled people accessing hospital care. It is important for hospital staff to listen to the perspectives of disabled people about the provision of reasonable adjustments and make improvements as necessary. Hospital staff could also do more to share good practice in relation to the provision of reasonable adjustments to effectively inspire and embed positive change.

## Additional file

Additional file 1: Interview Topic Guide. (DOCX 18 kb)

## Abbreviations

MRI scan: Magnetic resonance imaging scan
NHS: National Health Service

## Footnotes

1 In this paper, we have used the term ‘disabled person’ rather than ‘person with a disability’. While we appreciate that international readers may prefer terminology acknowledging the person first, the UK interpretation of the social model of disability regards ‘disability’ as a form of social oppression which individuals experience in society. This interpretation argues that a disabled person is not a ‘person with a disability’ as disability is not a characteristic that is part of, or owned by, the individual themselves, but is instead shaped and changed by how society is experienced.

2 While we have used the term ‘reasonable adjustments’ in this paper to reflect the terminology of the Equality Act 2010, international readers may be more familiar with the term ‘reasonable accommodations’.
