# Supplementary material for: Disabled people’s experiences of accessing reasonable adjustments in hospitals: a qualitative study
Source: BMC Health Serv Res. 2018 Dec 4;18:931. doi: 10.1186/s12913-018-3757-7 (PMC6278140; doi:10.1186/s12913-018-3757-7)
Supplement: Supplementary file 1 — Interview Topic Guide. (DOCX 18 kb) [file 12913_2018_3757_MOESM1_ESM.docx]

**Interview Topic Guide**

**Before Interview**

1. Do you identify as a disabled person?
2. Have you attended hospital in the last two years?
3. Where did you hear about our study?
4. Demographics: Age, Gender, Ethnicity, Geographical region of hospital.

**Intro:**

Hospitals in the UK have a responsibility to make changes to services in order to best meet the needs of disabled people, to ensure that they receive the same quality of healthcare that non-disabled people receive. These changes to services are called ‘reasonable adjustments’.

The purpose of this interview is to explore your experiences of hospitals, and whether hospital staff made changes to services to suit your needs. Specifically, we are interested in how hospitals make changes to meet your needs as a disabled person, rather than needs that are common to all patients regardless of disability (e.g. food and drink).

I will ask you to think about one hospital visit/stay you have had in the last two years.

Shortly, I’m going to ask you to tell me about your chosen hospital visit/stay, and that I may want to follow up some of what you say with some additional questions. If you feel you have said everything you want to say, or do not think something is relevant, we can move on. We can also come back to a previous discussion later on if you think of something that you would like to share. Afterwards, I would like to have a broader discussion about the changes that hospitals could make for disabled people so that disabled people are not disadvantaged in accessing healthcare.

| **Topic** | **Possible questions to use** | **Prompt** |
| --- | --- | --- |
| **1. Factual/clarification information about the hospital visit being reported**  **2. Arrangements prior to hospital visit/attendance** | **Before**   1. First of all, can you tell me what hospital visit/stay do you want to talk about? 2. Can you tell me about before you went into hospital/ before your hospital visit: How did the hospital know what your needs as a disabled person were? | When was this hospital visit/stay?  Which department or departments did you visit/stay? For example, did you use accident and emergency, outpatients department?  Follow up: Which department did you visit/stay? For example, physiotherapy, eye care etc.  For example, did you have a meeting with hospital staff to discuss your needs?  Do you think your GP might have contacted the hospital to discuss your needs?  Did you have a preadmission visit at which your needs were discussed and particular arrangements made for you?  Are there other changes that you can think of that the hospital made for you before you came to hospital?    What worked well, and what could have been done differently?  Why did X work well? Why didn’t X work well? How could X be changed? |
| **3. Meeting the person’s needs in hospital** | **During**  During your hospital stay/visit, how did the hospital/hospital staff met your needs as a disabled person?  Were these changes made especially for you, or do you know whether they were also for other patients? | What worked well, and what could have been done differently?  Why did X work well? Why didn’t X work well? How could X be changed? |
| **4. After the hospital visit/stay** | **Discharge/Afterwards**  After your hospital stay/visit, how did the hospital/hospital staff met your needs as a disabled person?  Were these changes made especially for you, or do you know whether they were also for other patients? | For example, did the hospital give you any information to take away with you? Are there other changes that you can think of that the hospital made for you after you left hospital?  What worked well, and what could have been done differently?  Why did X work well? Why didn’t X work well? How could X be changed? |
| **Any other comments about hospital visit/stay**  **Ideas and suggestions for future improvements** | **Review**  We have finished talking about your hospital visit now; is there anything else you would like to add before we move on? We can revisit this again if you think of something later on.  We are also interested your ideas and suggestions for how hospitals could made changes to/improve their care practices based on the experiences of disabled people.   1. Have you/your family/support worker provided feedback to the hospital to about how your needs were met? Has the hospital made any changes to their service(s)? 2. What changes do you think your hospital could make to their service(s) to help disabled people? 3. Is there anything else that you have thought about that you would like to talk about? |  |
